# HIV and Lacaziosis, Brazil

**DOI:** 10.3201/eid1203.051426

**Published:** 2006-03

**Authors:** Marilia B. Xavier, Marcia M.R. Ferreira, Juarez A.S. Quaresma, Arival C. de Brito

**Affiliations:** * Federal do Para University, Belem, Para, Brazil

**Keywords:** HIV, Lacazia loboi, mycosis, AIDS, Jorge Lobo disease, lacaziosis, letter

**To the Editor:** Jorge Lobo disease (lacaziosis) is a chronic deep mycosis for which prognosis is good in terms of survival but unclear in terms of regression of the lesions (*1*). No involvement of internal organs or mucous membranes is observed. The causative agent is *Lacazia loboi* (*2*), a fungus of uncertain phylogeny, which causes an inflammatory infiltrate accompanied by the formation of a granuloma in which giant cells phagocytose a larger number of fungi (*3**,**4*). Pecher and Funchs suggested that patients with lacaziosis have a cellular immunodeficiency (*5*). The disease is more frequent in men and persons 21–40 years of age. It is found exclusively in Latin America; only 1 case has been diagnosed in Europe, and that was due to accidental contamination with material from a dolphin (*4*).

Trauma and injuries or sites of insect bites facilitate penetration of the fungus. Lesion progression is slow, with new lesions arising by contiguity with other lesions or through the lymphatic route (*6**,**7*). Clinically, lacaziosis manifests as keloidal lesions of solid consistency and variable size that contain small scales and crusts (*6*). The lesions are most frequently located in the auricle and on the upper and lower limbs. Cutaneous dissemination of the disease is observed in a relatively small number of cases. We describe a patient with Jorge Lobo disease.

The patient was a 59-year-old man, a storeroom employee, who was seen at the Tropical Medical Center in Belem, Brazil, in April 2004. A papula had developed near his right knee in 1992 after a wood splinter had penetrated the skin. The lesion increased in size, and a histopathologic diagnosis of Jorge Lobo disease was made. The lesion was then surgically removed. Approximately 2 years later, the lesion recurred. The patient then went to a dermatology service and was treated with clofazimine, after which the lesion disappeared. However, the lesion reappeared 1 year later.

HIV serologic analysis was performed in 2002, and the results were positive. The patient then began treatment for HIV infection. He is currently being monitored at the specialized referral unit in Belem. He does not have any opportunistic infections and is not taking any antiretroviral drugs. The patient came to the dermatology service of the Tropical Medical Center, where dermatologic and histopathologic examinations were conducted and CD cell counts and HIV viral load were measured. Dermatologic aspects of the lesion included an erythematous-infiltrated, hypertrophic plaque with a verrucous surface ≈4 cm long in the distal third of the medical aspect of the right thigh (Figure). A punch biopsy specimen of brown smooth skin 0.35 cm in diameter in an epidermal disk was fixed in formalin. Microscopy of skin sections containing epidermis showed compact keratinization, parakeratotic foci, and irregular hyperplasia with a pseudoepitheliomatous area. A highly dense, nodular, diffuse inflammatory infiltrate was observed at all levels of the dermis. It consisted of macrophages and numerous multinucleated cells, most of them of the foreign body type. Fibroplasia was also noted. Abundant, round parasitic elements surrounded by a double membrane and containing a basophilic nucleus were found in tissues, as well as other anucleated, intracellular, and free parasites that formed chains of >2 cells (Figure A1). Jorge Lobo disease was diagnosed. Laboratory results showed 146 CD4 cells/μL, 251 CD8 cells/μL, a CD4:CD8 ratio of 0.42, and 60,000 copies of HIV viral RNA/mL.

Since a cytotoxic response is observed in Jorge Lobo disease (*7*), HIV infection may increase the susceptibility to infection with *L. loboi*. Patients with AIDS show a predisposition to diverse fungal infections that classically affect different organs and systems. An association between Jorge Lobo disease and AIDS has not been reported. However, since Jorge Lobo disease is restricted to specific areas of the world and the number of AIDS cases is increasing, especially in Latin America, a possible correlation between HIV infection and Jorge Lobo disease should be considered because of the associated cellular immunodeficiency.

The patient showed no signs of other opportunistic infections classically associated with AIDS, and he was not taking any antiretroviral drugs. His initial infection manifested as cutaneous lesions that occur in Jorge Lobo disease. Despite the cellular immunodeficiency, we did not observe atypical dissemination of the lesions. Further studies should be conducted to evaluate the relationship between the cellular immunosuppression of AIDS and secondary infection with *L. loboi*. In addition, epidemiologic studies are needed to determine the association of AIDS with Jorge Lobo disease.

**Figure:**
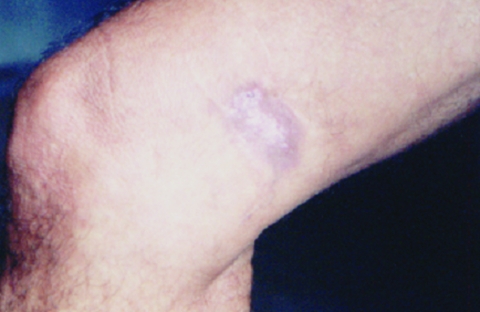
Erythematous-infiltrated, hypertrophic plaque with a verrucous surface ≈4 cm long in the distal third of the medical aspect of the right thigh of the patient.

## Figures and Tables

**Figure A1:**
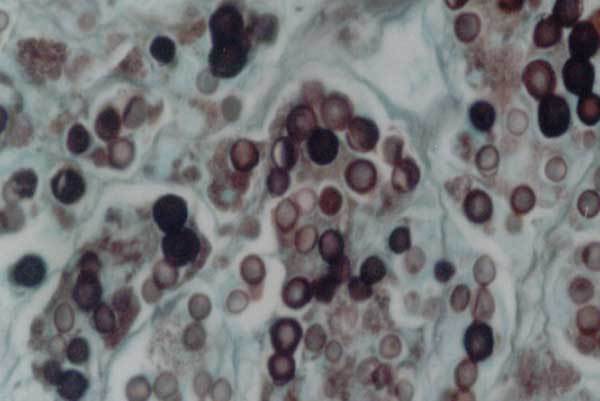
Abundant round parasitic elements in skin tissue of the patient surrounded by a double membrane and containing a basophilic nucleus. Magnification 400×.
